# Comparison of two sedation protocols for long electroretinography in horses using the Koijman electrode

**DOI:** 10.1186/s12917-023-03654-9

**Published:** 2023-08-04

**Authors:** Corradini Ignacio, López-Murcia María del Mar, Barba Marta, Zebarjadian Sina, Rodilla Vicent, Mayordomo-Febrer Aloma

**Affiliations:** 1 Departamento de Medicina y Cirugía Animal, Facultad de Veterinaria, Universidad Cardenal Herrera-CEU, CEU Universities, Tirant lo Blanc, 7, Alfara del Patriarca, Valencia, 46115 Spain; 2 School of Veterinary Medicine and Science, University of Nottingham, Sutton Bonington Campus, Nr Loughborough, LE12 5RD England, UK; 3 Departmento de Farmacia, Facultad de Ciencias de la Salud, Instituto de Ciencias Biomédicas, Universidad Cardenal Herrera-CEU, CEU Universities, Santiago Ramón y Cajal, s/n., Alfara del Patriarca, Valencia, 46115 Spain

**Keywords:** Long electroretinography, Horse, Detomidine, Butorphanol, Koijman electrode

## Abstract

**Background:**

In modern times, horses are utilized not only for labour and transportation purposes but also for recreational activities such as competition and pleasure riding. In these various pursuits, the role of vision plays a crucial role. Electroretinography is the most used test to diagnose diseases of the retinal outer segment. There is a wide variety of devices to perform the electroretinography differing one from each other in the corneal electrode and the light stimulation. The Koijman electrode has been tested in dogs but not in horses. The main purpose of this study was to compare electroretinography parameters from horses sedated with detomidine alone or in combination with butorphanol, during a standardized protocol using the Koijman electrode and RETI-port® system. Seven mares were allocated to the detomidine and detomidine plus butorphanol group in a randomised, controlled, crossover study. Friedman and Willcoxon-signed ranked tests were used to compare the electroretinogram parameters. A Student’s t-test was used to compare differences in the number of artefacts to valid values ratio obtained under both sedation protocols.

**Results:**

Dark adaptation peaked after 16 min under scotopic conditions in both groups. No significant differences in electroretinogram parameters between groups were observed. During the mixed rod and cone response evaluation under scotopic conditions, all mares made a movement of the head resulting in a high number of artefacts. The detomidine plus butorphanol group showed a non-significant tendency to have fewer artefacts and a longer duration of sedation compared to the detomidine group.

**Conclusions:**

Detomidine alone or combined with butorphanol may be suitable to use Koijman electrode and the RETI-port® to perform a standardized long protocol in horses with some adaptations.

## Background

The electrodiagnostic test of the eye allows for non-invasive evaluation of the visual pathways from the retina to the cortex. In veterinary practice, the electroretinogram (ERG), also called flash ERG, is widely used to assess the function of the outer layers of the retina and retinal pigment epithelium [1, 2]. The ERG represents the electrical response generated by the retina when stimulated by a brief light stimulus. Compared to ophthalmoscopy, ERG provides a more objective assessment of retinal function. In horses, ERG is routinely used prior to cataract surgery or for objective evaluation of diseases that affect the retina without changes in the ocular fundus examination [3–6]. It can also be utilised to quantify the functional damage of the retina when lesions are observed by indirect ophthalmoscopy [7]. Electroretinography is used in the assessment of other ocular diseases in horses such as equine recurrent uveitis or glaucoma[5].

Guidelines for clinical ERG evaluation have been published for dogs and humans [8]. However, there are few published studies evaluating the normal ERG parameters and its clinical use in horses [1, 5, 9–11].

Electroretinographic examination should ideally be performed under general anaesthesia. However, due to the increased intrinsic risks related to general anaesthesia in horses, standing ERG evaluation under sedation is preferred [12]. Sedation in horses during ERG procedure is essential to prevent free movement artefacts as well as to guarantee a safe access to the head and eyes and to protect the equipment [13]. Alpha-2 agonists and opioids are commonly used for sedation and analgesia in horses [14]. These drugs are known to alter different physiological parameters and certain aspects of ophthalmic examination and ERG in horses and other species [15–25]. Sedation protocols for ERG examination in horses should provide deeper and longer time duration of sedation compared to those used for routine ophthalmic examination [1]. The use of different alpha-2 agonists to perform the electroretinography have been published in horses [1, 7, 9, 10, 13, 26]. However, these do not eliminate all head movements during the procedure, which makes the examination less reliable and more time consuming [12]. Good muscle relaxation, tolerance to electrode placement and absence of head movements are of paramount importance in order to obtain good quality ERG recordings. Unfortunately, this is not always achieved by using only one class of drug [27].

There is evidence of the benefits of combining alpha-2 agonists with opioids (with or without peribulbar anaesthesia) to improve analgesia and sedation during some ophthalmological procedures in different animal species [28–30]. It has also been shown that the combination of detomidine (D) and butorphanol (B) provides better and safer sedation in horses compared to D alone either for non-painful and painful procedures in standing horses [15, 19].

Many ERG devices are commercially available on the veterinary market, with the primary distinctions among them lying in the light stimulation source and type, as well as the recording system employed. A full-field ERG stimulator (Ganzfeld dome) is preferred in order to obtain an overall response from the retina [2]. However, this has several disadvantages including its high cost, large in size and difficulties in correctly positioning the horse’s head for its use. There is one ERG device which has a contact lens electrode with the light emitting diode (LED) incorporated into the eyepiece. This electrode, called Koijman electrode, has been widely evaluated and used in dogs [31–34], but not in horses. The Koijman electrode is very light and has the additional advantage of keeping a constant distance between the flash and the retinal surface. These characteristics could present a simple way to perform ERG in the horse.

This study aimed to corroborate the usefulness of the Koijman electrode to obtain ERG readings in horses and to compare the ERG parameters obtained with the Koijman electrode using two sedation protocols: D alone or detomidine plus butorphanol (DB).

## Results

A level of sedation, characterised by lowering of the head, dropping of the lower lip, ears and upper eyelids was achieved on all animals in both groups. In addition, good akinesia of the upper eyelid was achieved on all eyes after the auriculopalpebral nerve block was performed. No facial or head tremors were observed in any of the study subjects in the DB group.

### Rod’s adaptation to low intensity flash under scotopic conditions

**Fig. 1:**
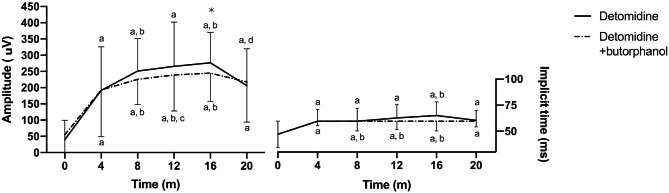
Median and interquartile range [IQR] amplitude and implicit time (IT) of *b* wave during the dark adaptation period in both anaesthetic protocols. *p < 0.05

The implicit time (IT) and amplitude values for the *b* wave at different times during the 20 min dark adaptation period of both anaesthetic protocols (median and interquartile range [IQR]) are shown in Table 1. Dark adaptation peaked at 16 min after initiation of scotopic conditions (T_16_). The amplitude of *b* wave was significantly lower (*p* = 0.041) at T_20_ compared to T_16_ in the D group (206.5 [132–341] µV vs. 276.5 [158–373] µV respectively) (Fig. 1). No significant differences in ERG parameters between both sedation protocols were observed (*p* = 0.14).

**Table 1:** Median and interquartile range [IQR] data of latency (ms) and amplitude (µV) obtained in the detomidine (D) and detomidine plus butorphanol (DB) groups.

		Adaptation-Scotopic						Scotopic mix		Photopic	
		*b* wave						*a* wave	*b* wave	*a* wave	*b* wave
		T_0_	T_4_	T_8_	T_12_	T_16_	T_20_				
D	Latency	47.1 [12.6]	59.6 [11.3]	59.6 [12.2]	62.7 [13.0]	65.1 [13.0]	60.4 [9.4]	14.7 [2.5]	57.8 [3.9]	12.9 [2.3]	27.1 [1.1]
	Amplitude	39.9 [53.7]	191.5 [134.0]	251 [99.7]	265.5 [136.5]	276.5 [94.0]	206.5 [113.5]	74.3 [70.3]	278.0 [148.0]	18.7 [22.6]	134.0 [53.0]
DB	Latency	47.1 [12.8]	59.6 [3.9]	59.6 [9.4]	59.6 [7.9]	59.6 [9.4]	59.6 [5.5]	15.7 [1.00]	59.8 [11.8]	13.5 [2.3]	27.6 [1.1]
	Amplitude	54.9 [66.0]	193 [144.1]	225.5 [78.0]	239 [111.0]	244.5 [87.7]	217.5 [123.5]	95.1 [54.3]	219.0 [216.0]	15.4 [18.7]	145.0 [32.0]

### Mixed rod and cone’s response under scotopic conditions

**Fig. 2:**
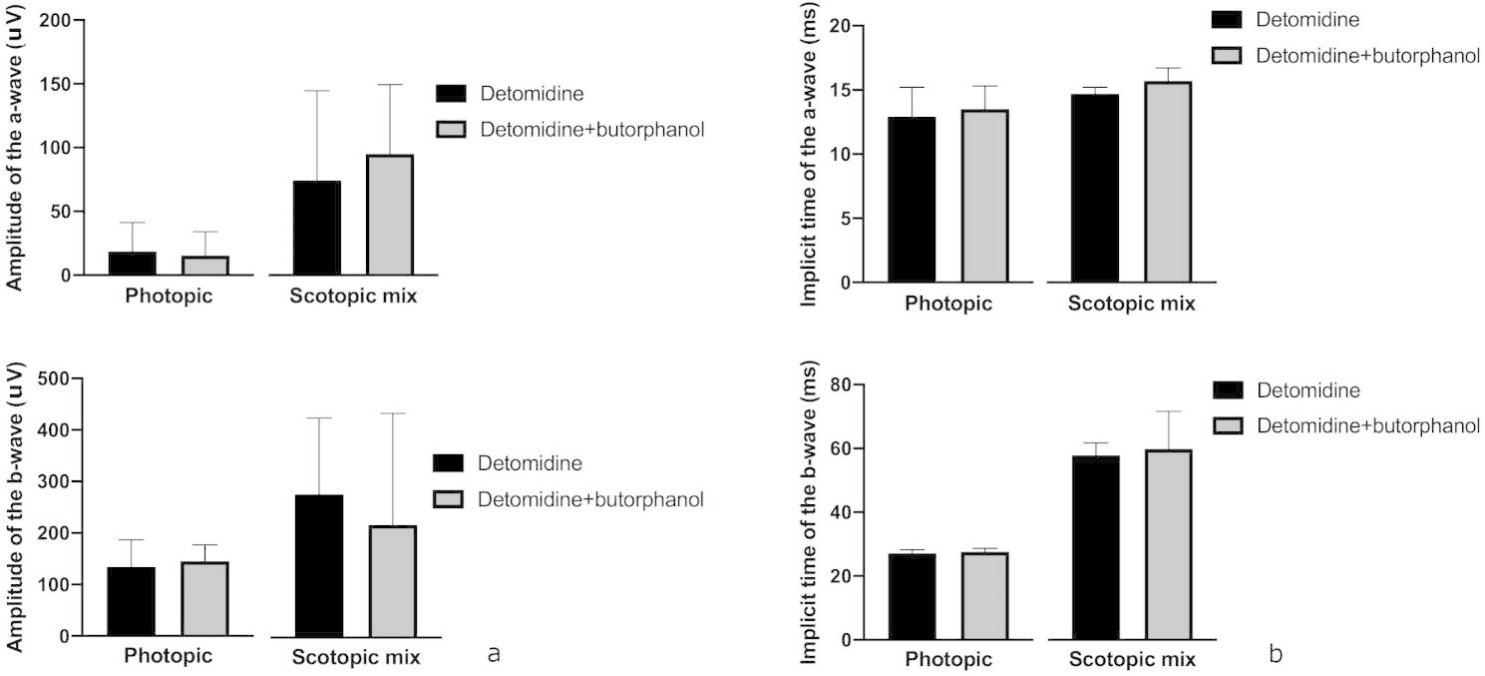
(**a**) Median and interquartile range [IQR] amplitude and (**b**) implicit time (IT) of *a* and *b* wave during the scotopic mix (mix rod-cone stimulation) and photopic step in both anaesthetic protocols

The median [IQR] *a* wave IT and amplitude for the mixed rode-cone response under scotopic conditions (scotopic-mix) were 14.7 [5.9–17.6] ms, 74.3 [9.18–109] µV and 15.7 [13.7–16.7] ms, 95.1 [25.1–159] µV for the D and DB groups respectively. The median [IQR] *b* wave IT and amplitude for the mixed rode-cone response were 57.8 [36.3–64.7] ms, 278.0 [94.2–454] µV and 59.8 [36.3–59.8] ms, 219.0 [92.9–443] µV for the D and DB groups respectively (Fig. 2). No significant differences in ERG parameters between both sedation protocols were observed (*p* = 0.39).

### Cone and rod response to high intensity flash under photopic conditions

The median [IQR] *a* wave IT and amplitude under photopic conditions were 12.9 [8.2–16.5] ms, 18.7 [3-43.3] µV and 13.5 [7.6–14.7] ms, 15.4 [3.4–39.9] µV for the D and DB groups respectively. The median [IQR] *b* wave IT and amplitude under photopic conditions were 27.1 [25.3–29.4] ms, 134 [29.6–196] µV and 27.6 [26.5–44.7] ms, 145 [71–200] µV for the D and DB groups respectively. No significant differences in ERG parameters between both sedation protocols were observed (*p* = 0.31).

### Incidents during the procedure, comparison of correct values, artefacts, and number of repeated sedations between both protocols

**Fig. 3:**
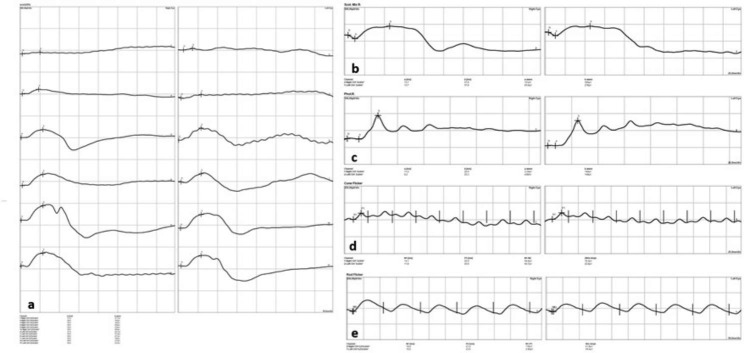
Representative electroretinography (ERG) obtained with detomidine (D) protocol in both eyes. (**a**) dark adaptation, (**b**) mix rod-cone response, (**c**) photopic response, (**d** and **e**) cone and rod flicker respectively. (N: flash onset, a: *a* wave, b: *b* wave, P: oscillatory potentials latencies)

**Fig. 4:**
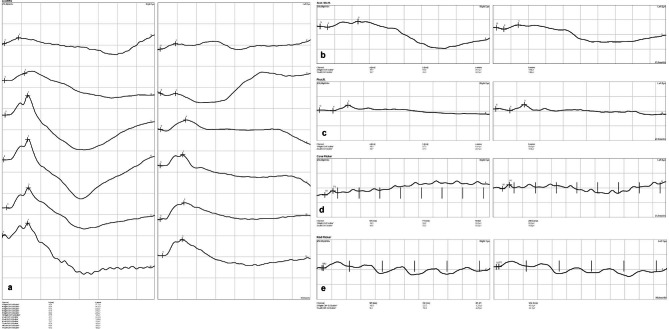
Representative electroretinography (ERG) obtained with detomidine plus butorphanol (DB) protocol in both eyes. (**a**) dark adaptation, (**b**) mix rod-cone response (**c**) photopic response, (**d** and **e**) cone and rod flicker respectively. (N: flash onset, a: *a* wave, b: *b* wave, P: oscillatory potentials latencies)

There were no complications related to sedation protocols, catheter placement or auriculopalpebral blocking in any of the mares examined. The fluorescein staining performed at the end of the procedures was negative in all patients. Electroretinographies were carried out without incidents in all steps obtaining adequate curve readings (Figs. 3 and 4). However, during the mixed rod and cone response evaluation under scotopic conditions, while using the 3 cd s m^− 2^ flash, all mares made an exaggerated head retreat response. This jerky movement of the head resulted in a very high number of artefacts; in fact, valid recordings were not obtained from 4/14 eyes (all from different mares) in the D group, and from 3/14 eyes (also from different mares) in the group DB.

The mean number of artefacts (measurements rejected automatically by the ERG system) was 103.6 for the D protocol and 94.8 for the DB sedation protocol. There were a lower number of artefacts when D and B were combined, but this difference was not statistically significant. The ratio of artefacts/valid values obtained at 3 cd s m^− 2^ was 4.86, indicating that for each valid value, approximately 5 artefacts were recorded. For all other steps of the ERG procedure this ratio was always below 1. The number of repeated sedations required for each protocol did not differ significantly either but was lower in the DB group.

### Post-hoc power calculations for each individual ERG parameter

Post-hoc power analysis showed that a power equal to or above 0.8 was reached for 12 of the 20 variables measured, by using an N = 7 animals. A power of over 0.8 was calculated for critical parameters such as IT and amplitude of *b* wave under scotopic conditions at T_16_ and T_20_, a and *b* wave IT and amplitude during rod and cone response evaluation, and *a* wave IT and *b* wave amplitude during photopic conditions. The required N for the remaining 8 parameters would have ranged from 24 to over 100 animals in the experiment.

## Discussion

The present study shows that there are no significant differences in ERG variables, using the Koijman electrode, in adult mares sedated with either D or a combination of DB.

Studies evaluating the effects of sedation on ERG in horses report the use of alpha-2 agonists alone [3, 13], generally being D the drug of choice [1, 7, 9, 10]. Butorphanol is a *k*-agonist opioid that is commonly used for sedation and for premedication in horses prior to general anaesthesia. The results of this study suggest that D may be insufficient for some individuals that undergo the complete European College of Veterinary Ophthalmology (ECVO) protocol. The RETI-port® adapted ECVO ERG protocol requires a total of 24 values per eye (48 per horse) for the rod response under scotopic conditions (dark adaptation period), 4 values per eye for the combined rod-cone response (scotopic mix function) under scotopic conditions and a total of 24 values per eye under photopic conditions. In this study, there was a tendency for improved efficiency (less artefacts) during the ERG procedure under sedation with DB (94.8 artefacts) compared to D alone (103.6 artefacts); however, these differences were not statistically significant.

Electroretinographic examination should ideally be performed under general anaesthesia. However, anaesthesia-associated mortality in healthy horses is estimated to be much greater than in humans and other domestic animals. Complications include cardiac arrest, fractures or myopathy associated to the anaesthesia, post-anaesthetic myelopathy and neuropathies, among other complications [12, 35].

Drugs used for sedation and anaesthesia in animals can affect the results of ophthalmic examination; for instance, differences in tear production and intraocular pressure have been documented in experimental animals, dogs and horses given different sedatives and anaesthesia [16, 21, 22, 25, 36, 37]. According to several studies, electroretinography parameters can also be altered by the type of sedation and anaesthetic protocol [38–40]. However, no significant differences in *a* and *b* wave complexes were observed in a study comparing 4 different alpha-2 agonists in horses [13].

The effects of a combined sedation protocol using alpha-2 agonists with B on ERG parameters has not been previously evaluated in horses. In our study, this combination did not affect significantly ERG parameters compared to sedation with D alone.

It was difficult to obtain full recordings during the scotopic-mix step (rod-cone response to a high intensity flash of 3 cd s m-^2^, in the dark) with either protocol. In this step, many mares reacted by jerking their heads and generating an elevated number of artefacts. This observation has not been reported in previous studies utilising the same ERG protocol in D sedated horses. This could be due to the utilization of a distinct active electrode (ERG-jet™) and light source (mini-Ganzfeld electroretinographic unit) in other studies [7, 10]. It may be necessary to re-evaluate the flash intensity used during ERG when utilising the RETI-port® and the Koijman electrode. Alternatively, testing different sedation protocols to deepen the level of sedation or exploring other sedatives agents could be considered.

Similar to the study by Church and Norman (2012), there was a high variability in the measurements of wave amplitudes between horses in this study. This was particularly evident for the amplitude of the *b* wave during scotopic conditions under both sedation protocols (Table 1). The *b* wave amplitudes ranged from 94.2 to 454 µV, being the largest variability seen at T_4_, T_20_ of the dark adaptation period, and at the scotopic mix step. The observed variations in ERG readings may have multiple contributing factors, such as variations in electrode placement, eye movement, pupillary dilation, different eye sizes and corneal curvature. In this sense, care was taken to adequately dilate the pupil, sedate, induce palpebral akinesia and provide appropriate head support to prevent additional sources of motion.

**Fig. 5:**
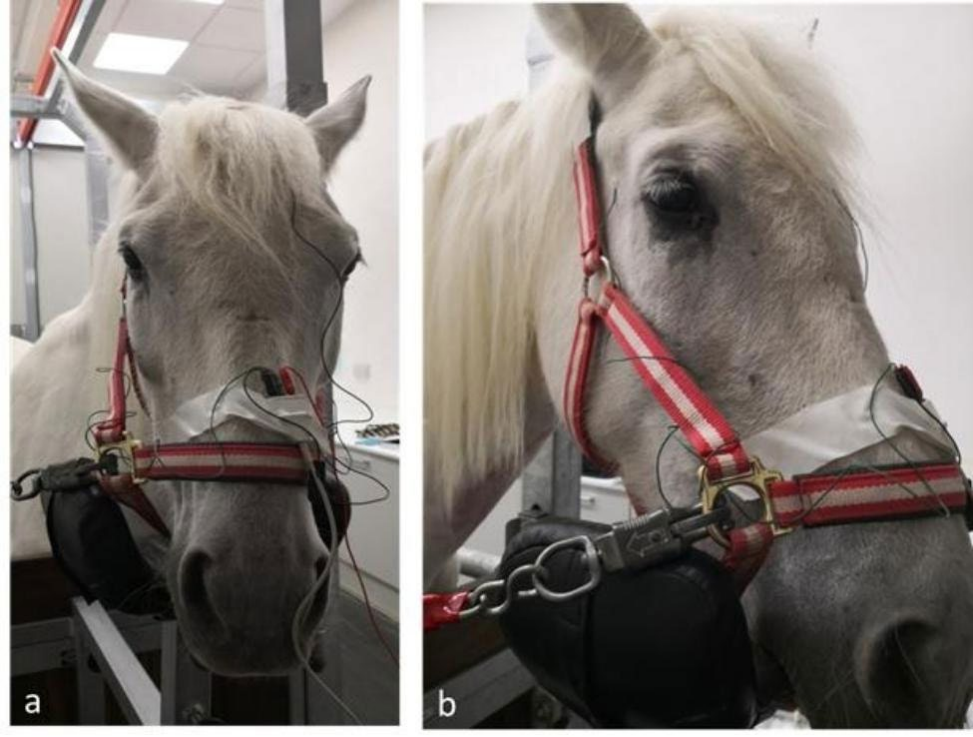
Electrode placement in a sedated mare resting the head over a padded head support. (**a**) ground electrode over the occipital bone. (**b**) reference electrode on the lateral canthus of the right eye

Peak adaptation to scotopic conditions occurred after 16 min in our study. For both protocols, *b* wave amplitudes gradually increased up to 16 min of dark adaptation being this difference statistically significant compared to T_0_ (*p* < 0.05) in both groups. The b-wave amplitude decreased at T_20_ of dark adaptation compared to T_16_; however, this decrease was only found to be statistically significant in the D group (Fig. 5). The IT gradually increased during the first 16 min of dark adaptation only in the D group (*p* < 0.05). Other studies have shown that dark adaptation takes longer in horses (30 min and even shown fluctuations with peaks in adaptation after 30 min to up to 1 h) [7, 9, 11]. This study was not originally designed to determine dark adaptation times; hence the dark adaptation time was limited to 20 min based on previous reports and the ECVO ERG protocol. The disparities observed here could be due to differences in pre-dark adaptation times, since horses were kept for several days in the same stalls under similar lighting conditions. In the examination room, they were kept under equal lighting conditions for 20 min and then the dark adaptation took place at 0 lx. During the 20 min prior to dark adaptation, luxmeters readings were higher than those reported for studies evaluating dark adaptation times in horses.

### Limitations of the study

Unfortunately, this study did not collect the data relating to the number or amplitude of head or body movements; hence, we were unable to carry out a statistical analysis comparing head and body movements between both sedation protocols. It is recommended that future studies focus on differences in head and body movements (using accelerometers, 2D or 3D head tracking, video-recording or similar technologies) when comparing different sedatives for ERG in horses.

Furthermore, in this study the evaluators were not blinded to the sedative being used which could have affected the perceived outcome and introduced a bias. It is also possible that the lack of statistical significance observed could be attributed to the relatively small sample size examined in this study. Regarding this matter, the sample size is relatively small to be able to reject the null hypothesis in 8 of the 20 ERG parameters recorded. However, it was found that the power of the test was in excess of 80% in critically important parameters such as *b* wave IT and amplitude under scotopic conditions at T_20_ and during mixed rod-cone response, as well as for some of the ERG parameters obtained during photopic conditions. Thus, increasing the sample size from 7 animals to more than 20 was not ethically, scientifically nor monetarily justified in the authors’ opinion.

Lastly, the Koijman electrode used in our study has a 17 mm ring that contacts directly with the cornea. Considering that the horse’s eye is large, compared to the dog, it is possible that the electrode may not stimulate the whole retina in a uniform fashion due to slightly different positioning between measurements. It is worth noting that another ring exists that may be suitable for use in larger animals (34 mm), but this ring was not available during this study. It is plausible that the larger ring might provide a steadier position over the cornea, as its diameter and curvature may adapt better to the horse’s eye. The authors were unable to find any studies comparing the accuracy of both rings for its use in horses along with the RETI-Port®. Further studies are needed to compare these two rings and to test for other potential sources of variability with this equipment arrangement.

## Conclusions

The Koijman electrode and the RETI-port® are devices that can be used to carry out the ECVO standard ERG long protocol. However, further studies are needed to determine if certain adaptations, such as lowering the flash intensity during the mixed rod-cone step, are needed. Future work comparing both, the large and small Koijman contact rings are necessary to confirm if this adaptor would reduce the variability of the data obtained with this equipment in horses.

Both anaesthetic protocols are suitable for performing ERG in horses with the Koijman electrode. The combination of D with B provided recordings with fewer artefacts than D alone; although, these findings were not statistically significant.

## Methods

### Animals and study design

Seven healthy crossbreed mares, ranging from 12 to 17 years old, from the teaching heard of the University CEU Cardenal Herrera, were enrolled in the study. All patients were weighed before entering the examination room and a complete physical examination was performed. Only healthy, non-pregnant mares were included in the study. Heart rate, respiratory rate, qualitative assessment of the mucous membranes, borborygmi and rectal temperature were monitored before sedation. However, no follow-up measurements were recorded as the effect of sedation on these physiological parameters was out of the scope of the present study.

Sample size was calculated based on the difference of *b* wave amplitude under scotopic conditions observed on a previous study comparing the effects of different alpha-2 agonist sedatives on ERG parameters in horses [13]. From those results, a standard effect size of 2.3 was calculated based on data showing that horses sedated with romifidine had an increased *b* wave amplitude of 358.5 + 78.4 µV compared to 171.2 + 84.0 µV in horses sedated with D [13]. Four animals were needed to detect a difference in *b* wave amplitude under scotopic conditions with a type I error of 0.05 and a power of 0.8 for a two-tailed Wilcoxon signed-rank test. To account for possible failure to obtain measurements, the procedure was performed in an additional three mares.

All animals were randomly assigned to the D group, positive control group, and DB group in a crossover study with a washout period of a minimum of 7 days. The evaluators were not blinded to the sedation being used. In the DB group, D was administered first, followed by the B three minutes later to prevent potential undesired side effects such as facial and head tremors induced by the B.

### Ophthalmic examination

The ocular examination included vision assessment (menace response, dazzle and pupillary light reflexes), evaluation of corneal and palpebral reflexes, slit-lamp examination of the anterior segment (Kowa SL-15®, Kowa Optimed, USA), intraocular pressure measurement using a rebound tonometer (TonoVet iCare®, Icare, Finland) and fluorescein staining to assess the integrity of the corneal epithelium. The ocular fundus was evaluated after pupillary dilation, necessary to carry out the ERG test, using the Panoptic ophthalmoscope (PanOptic®, WellchAllyn, United Kingdom) and the direct ophthalmoscope (WellchAllyn, United Kingdom).

### ERG preparation

All horses were stalled in the same hospital area under similar light conditions for a minimum of one week prior to the study. In addition, all procedures were performed in the same room, with the same light arrangements. To ensure that all animals were tested under the same light conditions, all ERG procedures were performed between 9:30 and 13:00 h. In addition, two luxmeters (Portable luxmeter,Urceri® and MT-30 Digital Lux Meter 200,00 lx) were used to guarantee similar lighting conditions during the preadaptation period and the different stages of ERG examination. One luxmeter was placed at the horse’s eye level, secured to the right front poll of the stocks, and the other luxmeter was placed on top of the ERG equipment to the left front of the stocks. Both luxmeters recorded 0 lx during the scotopic phase of ERGs.

Tropicamide (0.2 ml of a 1% solution, Colircusí Tropicamida® 10 mg/ml, Alcon, Spain) was applied to both eyes to induce pharmacological mydriasis every 5 min, until complete dilatation was achieved.

Sedation was administered through a 14-gauge teflon catheter placed on the left jugular vein, using one of the following sedation protocols: D (Sedaquick®, Fatro, Spain) (15 µg/kg) or D and B (Butomidor, Richter Pharma AG, Austria) (15 µg/kg for both products). If required, a rescue dose was administered (5 µg/kg of D for the D group, or 5 µg/kg of each of the drugs for the DB group).

An auriculopalpebral nerve block was performed bilaterally by injecting 1.5 ml of a 2% lidocaine solution (Lidocaína inj. 2%, B.Braun, Spain) after sedation.

To improve horses’ wellbeing and to provide additional stabilization of the head, a padded head support attached to the stock’s door was used.

### Electrode set-up and recording equipment

**Fig. 6:**
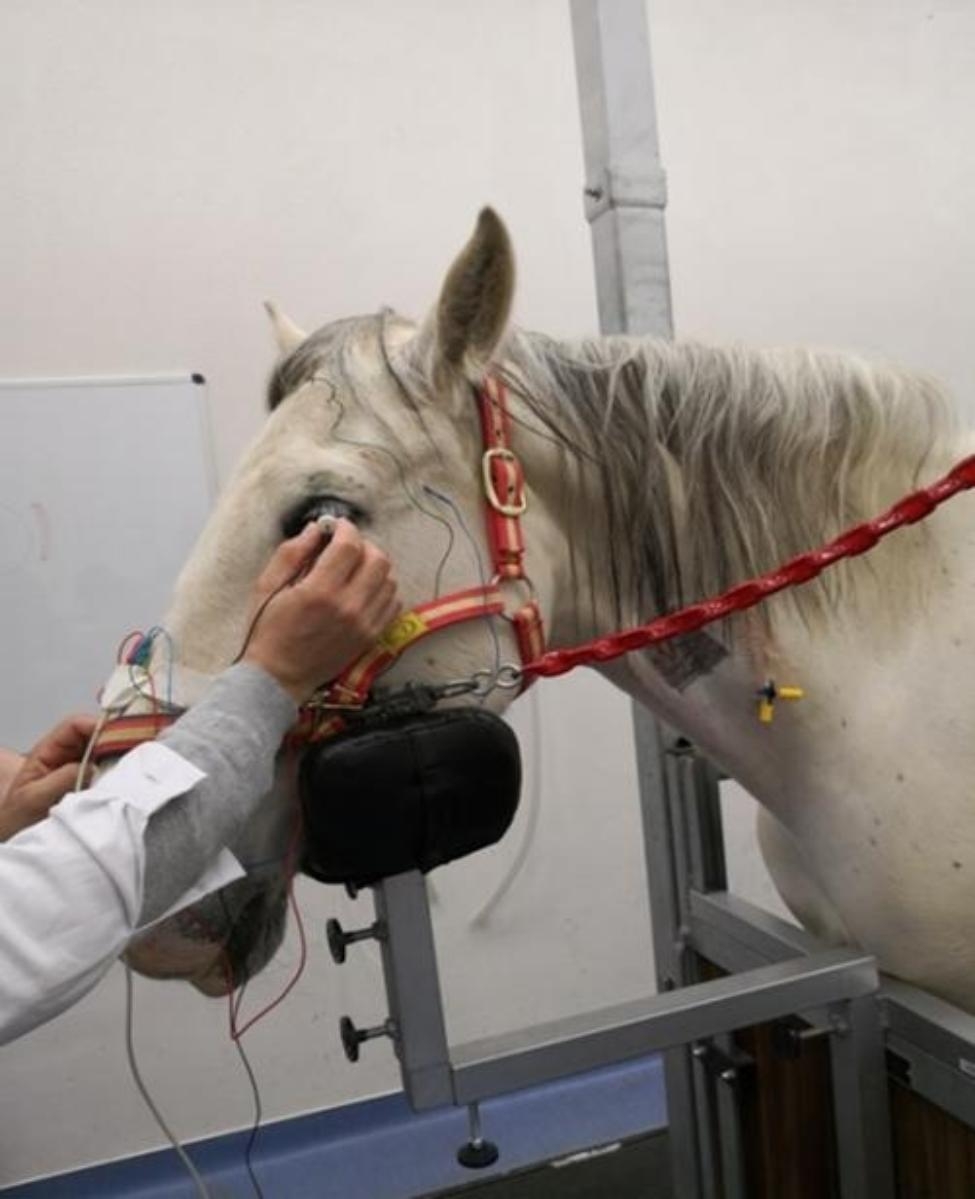
Koijman electrode positioned over the left cornea, just before to start the photopic stimulation

Titanium subdermal needle electrodes (Roland Consult, Germany) were placed subcutaneously 3 cm caudal to the lateral canthus of the eye being evaluated (reference electrode) and at a central location over the occipital bone (ground electrode) (Fig. 5). Both electrodes were secured to the halter with medical tape. A monocular LED contact electrode (Koijman electrode 17 mm contact ring, An-vision, Germany) was placed over the cornea (active electrode) and was gently held by the operator during the procedure (Fig. 6). Prior to electrode placement, 0.2 ml of topical anaesthetic (Colircusí anestésico doble®, Alcon, Spain) was applied to both eyes. To avoid damaging the corneal surface and to ensure good contact between the electrode and the cornea, a generous amount of lubricant gel (Lubrithal™, Dechra, Spain) was applied to the electrode as needed during the procedure. To ensure accurate delivery of flash duration and intensity, the manufacturer calibrated the flash of the unit prior to the start of the study.

### Testing protocol

Electrodes were connected to the RETI-port**®** ERG-system (Roland Consult, Germany) and impedance to ground was measured for both the active and the reference electrodes. The flash duration was set at 10 ms and the light intensities varied between 0.01 cd s m^− 2^ and 3 cd s m^− 2^ depending on the step of the protocol.

Scotopic and photopic ERGs were recorded, from both eyes of each mare, using a standard long ERG protocol (Table 2) following the guidelines for clinical ERG in the dog set by the ECVO. These guidelines were published by Narfstrom in 2002 and updated by Ekesten et al. in 2013 [2, 41].

**Table 2:** Summary of the European College of Veterinary Ophthalmology (ECVO) recommended electroretinography (ERG) protocol [1, 22]:

ERG step	Total duration	Cells evaluated	Flash intensity
Dark adaptation	20 min	Rod evaluation: at 0 (10 s after dark adaptation), 4, 8, 12, 16 and 20 min.	0.01 cd s m^-2^
Dark (scotopic mix)	Immediately after dark adaptation	Mixed rod-cone response evaluation with a single flash	3 cd s m^-2^
Light adaptation	10 min	Cone response evaluation with a single flash	3 cd s m^-2^
Flicker test	Immediately after light adaptation		3 cd s m^-2^

### Data analysis

Data were analysed using SPSS® 24.0.0.0 ^c^ by means of the Wilcoxon-signed rank (matched pairs) test to compare IT and amplitude of *a* and *b* waves between the D (n = 7) and DB (n = 7) groups. Assumptions of normal data distribution evaluated using the Shapiro-Wilk test, histograms and Q-Q plots were not met.

Data are presented as median and [IQR]. Multiple comparisons for repeated measures within treatment for both, IT and amplitude of *a* and *b* waves, were performed using the Friedman test. In addition, a Student’s t-test was used to compare differences in the number of artefacts, valid values, and the ratio of artefacts against valid values between sedation protocols as these data showed a normal distribution. Differences were considered statistically significant when *p* ≤ 0.05. The ratio of artefacts (invalid readings) to valid readings was calculated for all the steps of the protocol. Ratios over 1 meant that for each valid reading obtained more than 1 flash was necessary at any given step of the process. None of the data were excluded from the analysis.

Post-hoc power calculations were made for each parameter measured during the ERG protocol.

## Data Availability

All the data that support the findings of this study are available from the corresponding author upon reasonable request.

## List of Abbreviations

D: Detomidine
B: Butorphanol
DB: Detomidine and butorphanol
ECVO: European College of Veterinary Ophthalmology
ERG: Electroretinogram
IQR: Interquartile range
IT: Implicit time
LED: Light emitting diode
